# Thyroid ima artery embolization for the treatment of Graves’ disease and thyroid storm

**DOI:** 10.1016/j.radcr.2023.04.044

**Published:** 2023-05-28

**Authors:** Maximilian Bonnici, Connor Nevin, SoHyun Boo

**Affiliations:** a School of Medicine, West Virginia University, Charleston, WV, USA; b Department of Neuroradiology, West Virginia University Hospital, 1 Medical Center Drive, PO Box 8063, Morgantown, WV 26506, USA

**Keywords:** Thyroid ima artery, Embolization, Graves’ disease, Thyroid storm, Interventional radiology, Hyperthyroidism

## Abstract

Graves’ disease may be managed with thyroid embolization when other traditional strategies are less suitable. To date, no reports exist on the embolization of the thyroid ima artery for Graves’ disease or the outcomes of this procedure. Here, we present a safe and successful report of thyroid ima artery embolization in a neutropenic patient with thyroid storm. A 30-year-old female with a history of Graves’ disease presented to a community hospital with heat intolerance, anxiousness, tachycardia, and tachypnea. Further laboratory testing prompted the diagnosis of thyroid storm. Embolization of the thyroid was chosen for management because total thyroidectomy risked infection in the setting of methimazole-induced neutropenia. The patient's thyroid ima artery was significantly enlarged and had a large perfusion distribution across the thyroid. It was selected for embolization, along with the right superior thyroid artery, and successfully resulted in a 60% reduction of thyroid perfusion. No complications resulted from this procedure. Although the prevalence of patients with thyroid ima artery is low, this artery can be safely and effectively embolized for the management of Graves’ disease and thyroid storm.

## Introduction

The thyroid ima artery is a normal anatomic variant of thyroid vasculature found in 3.8% of the population [1]. This artery typically branches from the brachiocephalic trunk and is more commonly seen in fetuses, who are 4.5 times more likely to have a thyroid ima artery when compared to adults [1,2]. When present in utero, this artery is theorized to regress during the normal development of the thyroid [1]. Adults with this anatomic variant represent a unique subset of patients who may benefit from embolization in the setting of hyperthyroidism. The precipitation of thyroid storm in the setting of Graves’ disease can warrant embolization of thyroid vasculature when thionamides, radioactive iodine, and thyroidectomy are less tolerable. While thyroid embolization has been performed for the past 20 years, no cases of thyroid ima artery embolization have been reported in the treatment of Graves’ disease. In general, embolization of the thyroid ima artery is seldom reported but is safe and effective in managing mediastinal hematomas [3]. Here, we report the first known case of successful thyroid ima artery embolization to treat Graves’ disease in a patient with methimazole-induced neutropenia.

## Case presentation

A 30-year-old female with a recent diagnosis of Graves’ disease presented to an outside facility for worsening hyperthyroid symptoms. She described heat intolerance, myalgia, anxiety, and diarrhea after waking from a full 24 hours of sleep without interruption. Physical exam was notable for tachycardia, tachypnea, and proptosis. At this time, routine labs showed neutropenia with a white blood cell (WBC) count of 1.1 × 10^3^/µL. She had initially been prescribed propranolol and methimazole, but methimazole was discontinued at this time and she began filgrastim. Prophylactic antibiotics were also initiated. Ultrasonography at the outside facility identified a diffusely enlarged and hyperemic thyroid. She was then transferred to a tertiary care center and was started on thiamazole, propranolol, potassium iodide, and IV hydrocortisone once thyroid storm was suspected.

Evaluations from endocrinology and otolaryngology ruled against potassium iodide and radioactive iodine management for thyroid storm because of the lack of long-term efficacy and side effects. Thyroidectomy was additionally unfavorable in the setting of neutropenia which increased her risk of infection. As a result, interventional neuroradiology was consulted to perform thyroid embolization.

**Fig. 1:**
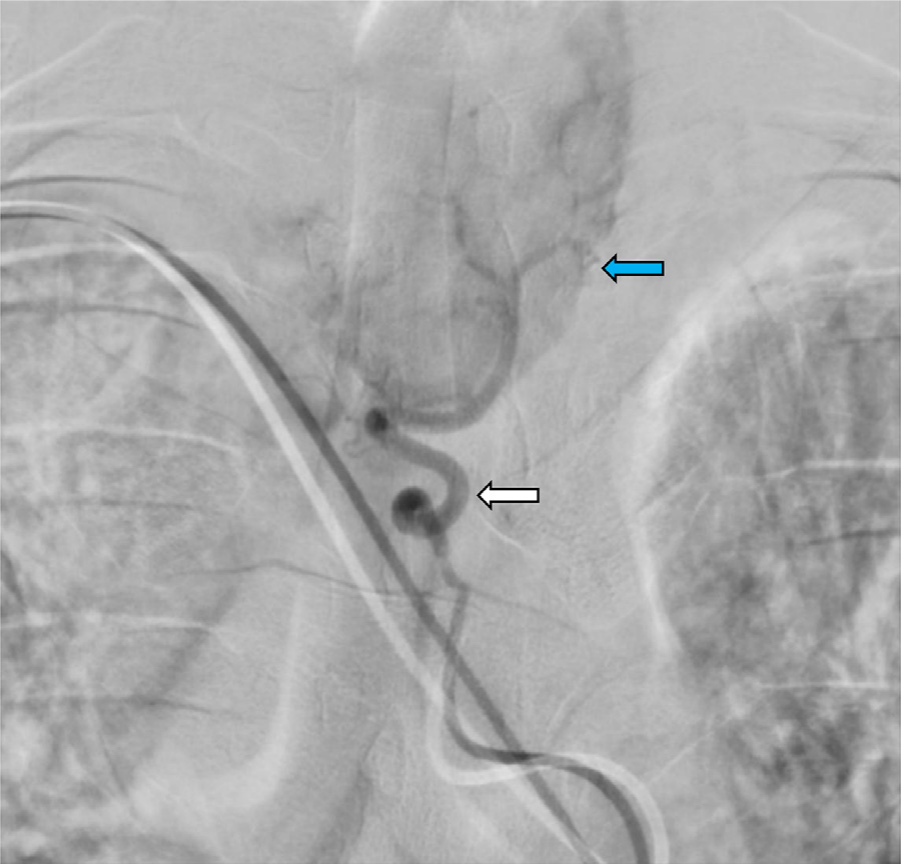
The thyroid ima artery (white arrow) can be seen ascending to the thyroid (blue arrow). This artery was enlarged and perfused well into the isthmus and the left upper and lower lobes of the thyroid, resulting in significant thyroid necrosis with embolization.

**Fig. 2:**
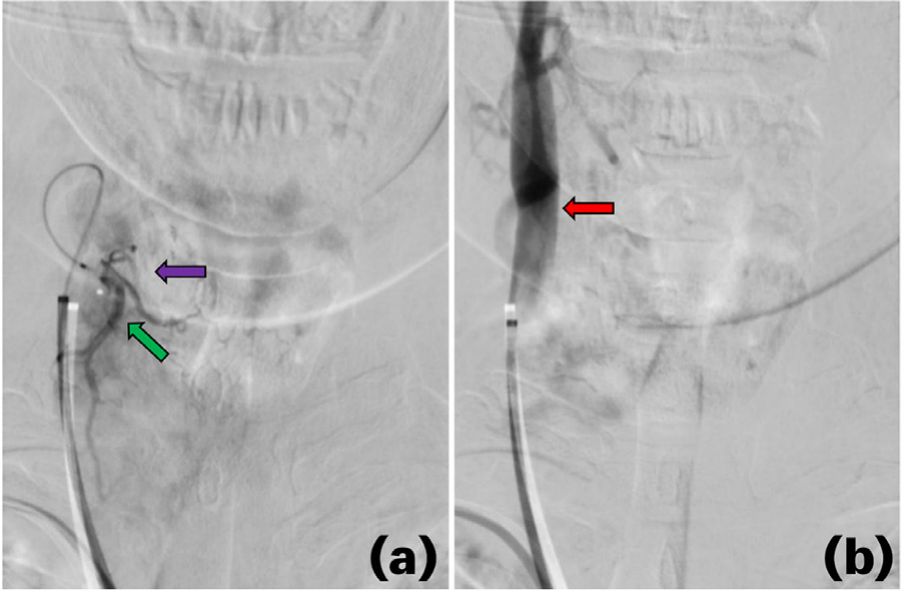
Angiography before (A) and after (B) embolization of the enlarged right superior thyroid artery (green arrow) which perfused into the right upper thyroid lobe (purple arrow). Embolization of the right superior thyroid artery inhibited thyroid perfusion, shunting more blood through the right carotid artery (red arrow).

**Fig. 3:**
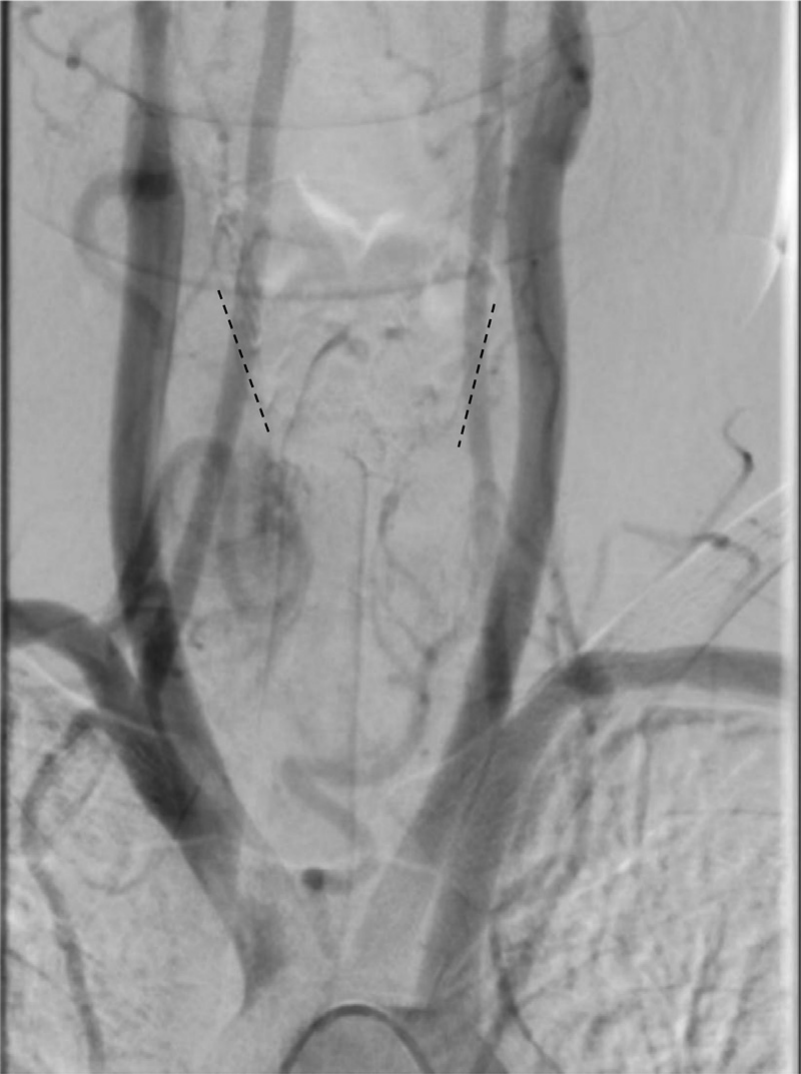
This arch aortogram displays a lack of perfusion to the thyroid tissue postembolization of the thyroid ima artery and the right superior thyroid artery. The dashed black lines outline the right and left borders of the thyroid.

During embolization, a micro-stick needle puncture and modified Seldinger technique were utilized, the radial artery was accessed, and a 6 French slender sheath was placed. Through this, a 5 French and Simmons 2 catheter were navigated over a 035″ Glidewire with fluoroscopy using the roadmap technique. Angiography of the right thyrocervical trunk demonstrated an enlarged right inferior thyroid artery (ITA) perfusing into the right inferior thyroid lobe. A Simmons 2 catheter was then selected into the left subclavian artery and identified a prominent left ITA draining into the left lower thyroid lobe and subsequently identified an enlarged thyroid ima artery perfusing into the isthmus as well as the left upper and lower thyroid lobes (Fig. 1). Angiography of the right common carotid artery showed the superior thyroid artery (STA) was enlarged and perfused into the right upper thyroid lobe. An XT 27 microcatheter was then navigated through a 6 French catheter to select the right STA for polyvinyl alcohol embolization (Fig. 2). The guide catheter was withdrawn and placed into the left common carotid artery where it demonstrated a normal-appearing left STA which arose at an acute angle from the carotid bifurcation. Right common femoral artery access was then obtained, and a Simmons 2 catheter was navigated for the selection of the thyroid ima artery. The thyroid ima artery was embolized with polyvinyl alcohol and the reverse roadmap technique was employed. All catheters were withdrawn, and a pigtail catheter was taken over the aortic arch for an arch aortogram. The findings demonstrated a 60% reduction in thyroid blush postembolization of the right STA and thyroid ima artery (Fig. 3).

The patient's symptoms of hyperthyroidism improved following embolization and remained in remission. One day postembolization, her WBC count normalized to 8.9 × 10^3^/µL and her neutrophils made up 61% of her total WBCs which was only 9% the day before. The patient was then eligible for total thyroidectomy and agreed to it the following day. Total thyroidectomy was successful but required reimplantation of the left superior parathyroid into the infrahyoid muscle because of its initial extraction. The patient had hypocalcemia prior to her admission from vitamin D deficiency. Her preoperative calcium levels were low at 7.0 mg/dL but did not decrease with parathyroid reimplantation. Calcitriol and levothyroxine were administered and her free T3 levels normalized several days later. Changes in her voice returned to baseline following the operation and her symptoms of hyperthyroidism had subsided. She was discharged 3 days postoperatively.

## Discussion

Thyroid embolization may be used for a diverse array of hyperthyroid etiologies, including hyperfunctioning goiters [4], Graves’ disease [5], and follicular thyroid carcinoma [6]. This technique is often selected because it safely promotes apoptotic factors which destroy the thyroid, reducing excessive thyroid hormone production [7]. Thyroid embolization is typically considered against the balance of thyroidectomy complications (Table 1) since both procedures carry unique risks [8,9]. Thyroidectomy carries a 20%-30% risk of hypocalcemia and a 5%-11% risk of recurrent laryngeal nerve injury which results in vocal hoarseness [8]. As seen with our patient, open thyroidectomy can be temporarily rejected because of the underlying risk of infection from neutropenia. While other treatment modalities of hyperthyroidism are well understood, thyroid embolization remains a novel therapy that is complicated by significant variations in thyroid vasculature [10]. Deviations in vessel anatomy, such as a distant point of origin, an acute angle of bifurcation, a slender lumen, or insignificant thyroid perfusion may help interventional radiologists decide against embolization of certain vessels in favor of others. When embolization is selected, it is 86% effective in reducing hyperthyroid nodules to a euthyroid state [9]. It has a 45% minor complication rate, consisting mostly of neck pain and subclinical hyperthyroidism, and a 4% major complication rate which includes symptomatic hyperthyroidism and groin hematomas [9].

**Table 1:** Thyroidectomy and thyroid embolization are indicated for similar causes of hyperthyroidism. The complications of both procedures are summarized.

Thyroidectomy vs thyroid embolization	
**Indications**	- Graves’ disease - Thyroid carcinoma - Toxic goiter - Recurrent goiter
**Thyroidectomy complications**	**Thyroid embolization complications**
- Infection - Hypocalcemia - Recurrent laryngeal nerve injury - Postoperative compressive hematoma	- Hoarseness - Neck pain - Subclinical hyperthyroidism - Symptomatic hyperthyroidism - Groin hematoma

Little is known about the efficacy of thyroid ima artery embolization given its scarcity in the adult population and the common practice of the STA and ITA occlusion. To our knowledge, only one published case comprehensively describes embolization of the thyroid ima artery whereby a traumatic injury to a 91-year-old man resulted in a mediastinal hematoma [3]. Gelatin particles and micrometal coils were employed for hemostasis and no complications related to catheterization were noted [3]. The procedure was successful and the patient recovered enough to be transferred to another hospital for rehabilitation [3]. In our case, embolization was employed to induce necrosis and apoptosis as opposed to hemostasis. The fact that our patient's enlarged thyroid ima artery supplied the left thyroid lobe and their enlarged right STA supplied the right upper thyroid lobe made embolization of both vessels optimal for diffuse thyroid destruction. Embolization of the left STA and ITA were less optimal since the STA angled off too acutely for embolization and the ITA only perfused into the left lower thyroid lobe.

## Conclusion

Endovascular embolization of the thyroid ima artery is safe and efficacious in reducing thyroid perfusion for Graves’ disease and the precipitation of thyroid storm when it is present and enlarged in the patient with Graves’ disease. No complications resulted from this procedure, and it provided sufficient time for the patient's leukopenia to resolve before total thyroidectomy.

## Author contributions

All authors have made substantial contributions to this manuscript.

## Ethical statement

All authors adhere to the ethical guidelines set forth by *Radiology Case Reports*. This study is exempt from the Institutional Review Board of West Virginia University.

## Patient consent

We confirm that written, informed consent for publication of our case was obtained from the patient.
